# Perceived regularity of a texture is influenced by the regularity of a surrounding texture

**DOI:** 10.1038/s41598-018-37631-2

**Published:** 2019-02-07

**Authors:** Hua-Chun Sun, Frederick A. A. Kingdom, Curtis L. Baker

**Affiliations:** McGill Vision Research, Department of Ophthalmology, McGill University, Montreal, Quebec Canada

## Abstract

Previous studies have shown that texture regularity is adaptable, and have suggested that texture regularity might be coded by the peakedness of the underlying spatial frequency distribution. Here we demonstrate the related phenomenon of simultaneous regularity contrast (SRC), in which the perceived regularity of a central texture is influenced by the regularity of a surrounding texture. We presented center-surround arrangements of textures and measured the perceived regularity of the centre, using a centre-only comparison stimulus and a 2AFC procedure. From the resulting psychometric functions the SRC was measured as the difference between test and comparison regularity at the PSE (point of subjective equality). Observers generally exhibited asymmetric bidirectional SRC, in that more regular surrounds decreased the perceived regularity of the centre by between 20–40%, while less regular surrounds increased the perceived regularity of the centre by about 10%. Consistent with previous studies, a wavelet spatial frequency (SF) analysis of the stimuli revealed that their SF distributions became sharper with increased regularity, and therefore that distribution statistics such as kurtosis and SF bandwidth might be used to code regularity.

## Introduction

Texture regularity, i.e. the degree of orderliness of element positions in a texture (henceforth, “regularity”) is a basic texture property. Regular textures consist of uniform patterns in a cyclical i.e. periodic arrangement^1,2^. Their opposites are textures composed of randomly positioned elements, maximizing their entropy and unpredictability^3^. Different degrees of regularity occur in both natural and artificial surfaces, and convey important information about their structure, etiology and biological function^4,5^. However we know little about how the visual system extracts regularity information.

A simple and commonly employed method to construct textures of varying regularity levels is to apply random perturbation (“jitter”) independently to the horizontal and vertical element positions in a notional lattice pattern of texture elements, e.g. dots - a larger range of the jitter gives more irregular textures^6–8^. Stimuli constructed using this method have been used to demonstrate that regularity is an adaptable dimension of vision. By measuring the perceived regularity of a pattern following adaptation to a pattern with a different degree of regularity, some studies have demonstrated a “regularity aftereffect”^8^ (or, as Yamada *et al*.^7^ put it, a “randomness aftereffect”). Specifically, perceived regularity is reduced after adapting to a more regular pattern^8^ and enhanced after adapting to a more irregular one^7^. Varying the element properties between adapt and test textures has revealed that regularity encoding operates on both luminance- as well as contrast-defined elements^8^, and that it is orientation selective but is insensitive to luminance polarity and retinal location^7^.

Note that regularity defined in relation to a lattice of element positions is different from the classical “symmetries” - mirror symmetry, radial symmetry, and translational symmetry^9–13^. Moreover, two of these, mirror- and radial-symmetry, are likely processed by a different class of mechanism to that mediating regularity and translational symmetry. Following on from Ouhnana *et al*.’s^8^ analysis of the Fourier composition of texture regularity, Jennings and Kingdom^14^ suggest that information about regularity and translational symmetry is likely carried by the Fourier amplitude spectrum whereas information about mirror- and radial-symmetry is likely carried by the Fourier phase spectrum. This idea is in keeping with the finding that regularity information is mostly preserved under Fourier phase scrambling^15^ and that the perception or discrimination of regularity does not require the encoding of the relative position of each texture element^16^. Such a scheme would enable regularity to be encoded rapidly and in parallel at an early stage of vision rather than via a higher level, time-consuming, serial process^17^. Consistent with the above findings/analyses, Ouhnana, *et al*.^8^, Yamada, *et al*.^7^ and Protonotarios *et al*.^18^ have suggested that the representation of regularity information is related to the peakedness of the distribution of oriented spatial-frequency-tuned filter responses.

Regularity has been found to influence the processing of other visual information. For example, perceived surface slant is greater for regular than irregular textures^1^. Regularity also affects numerosity perception by reducing clustering, which increases the estimated number of dot elements by 5~8%^19,20^. Regularity also plays an important role in texture segregation when shape discrimination is required - textures of regularly positioned elements lead to higher accuracy than textures of elements with random positions^21^. However, regular (equidistant) spacing of a series of continuous contours impairs contour detection, which lead to higher thresholds than random placement of contour elements^22^. This could be explained by element proximity, which is a much stronger cue for contour detection, because there are more close local pairings of elements in random placements than in regular ones^22^.

This study investigates the representation and encoding of regularity using a different approach – simultaneous contrast. Simultaneous contrast is a phenomenon in which perception of a visual property is altered by a surround having a different level of that property. It has been observed in many visual dimensions including spatial frequency^23^, contrast^24,25^, luminance^26^, orientation^27,28^, element size^29^ and texture density^30,31^. Here we use a simultaneous contrast approach to study regularity perception. We systematically vary the regularity levels of center and surround texture patterns and investigate how the perceived regularity of the center area changes with this manipulation, i.e., we measure simultaneous regularity contrast, or SRC. Previous studies on regularity adaptation utilized only a single test level, which was about half the inter-element distance in their texture patterns^7,8^ or just a few adaptation levels (higher, lower or the same as the test regularity)^7^. In this study we use a wide range of test and surround regularities, to obtain a more complete understanding of regularity processing.

## Methods

### Apparatus and Stimuli

Stimuli were generated using custom code in Matlab with the Psychophysics Toolbox^32–34^ and presented on a CRT monitor (Sony Trintron GDM-F520, 20 inches, 1600 × 1200 pixels, 85 Hz). Luminance was measured with an Optikon universal photometer, and linearized using Mcalibrator2^35^. The viewing distance was 85 cm.

The stimulus texture consisted of dark Gaussian blob elements generated using the Psychophysics Toolbox function ‘CreateProceduralGaussBlob’ (standard deviation sigma = 2.43 arcmin, minimum luminance 7 cd/m^2^, see Ouhnana, *et al*.^8^), presented on a mid-grey background (61 cd/m^2^). These elements were placed on a 54 × 54 notional grid (interelement distance 20.44 arcmin) for center and surround area, and the center-only grid was 8 × 8. Then each element was randomly jittered in horizontal and vertical positions, independently, within a jitter range based on conditions (see below). Overlapped elements were reassigned to other jitter locations within the same range. The regularity level is defined by the range of the jitter (uniform distribution), such that greater jitter range produces lower regularity.

Each trial consisted of two stimuli: a test texture and a match texture. Each texture pattern was presented in a circular center-surround arrangement, such that the diameter of the center area was 2.91 deg and the surround diameter was 17.46 deg. The test contained both center and surround textures, with their regularity levels manipulated separately. The match texture covered the center area only.

**Figure 1:**
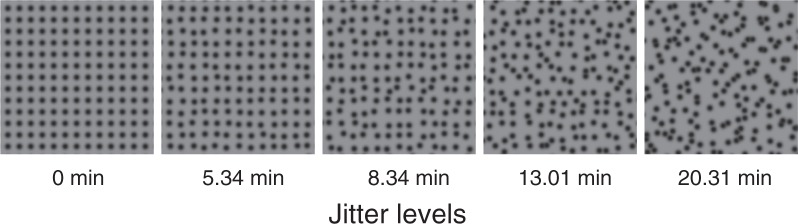
The primary texture regularity levels used in the experiment. The five regularity levels are used for test surrounds, and the middle three are used for test center. All the textures are based on the same square grid pattern (the first one). From left to right: by increasing the amount of jitter in each element position, the textures become more irregular^6^.

**Figure 2:**
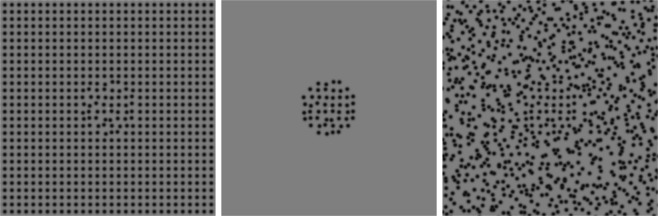
Examples of three test stimuli used in the experiment, all with the identical texture pattern in the center (8.34 jitter arcmin). From left to right: the test center was presented with the most regular surround (0 jitter arcmin), no-surround baseline (middle) and the least regular surround (20.31 jitter arcmin). When fixating at the center, the regularity of the center in the left appears less regular than others, and the right one appears more regular. This phenomenon is called simultaneous regularity contrast (SRC). Note that the surround region is cut-off here. In the formal experiment, the surround covers a circular area and its diameter is six times that of the center.

The center of the test stimulus was set at one of three regularity levels: 5.34, 8.34 or 13.01 jitter arcmin (Fig. 1, middle three examples). The surround of the test stimulus was set at one of five regularity levels: 0 (perfectly regular), 5.34, 8.34, 13.01, 20.31 jitter arcmin (Fig. 1), or set to “no-surround” (baseline condition). Altogether our test stimuli consisted of 3 center regularities × (5 surround regularities + 1 no-surround baseline) = 18 conditions in total. Examples of test stimuli, all with a center regularity of 8.34 jitter arcmin, are depicted in Fig. 2, with the most regular surround (0 jitter arcmin, Fig. 2 left), no-surround baseline (Fig. 2 middle) and a least regular surround (20.31 jitter arcmin, Fig. 2 right). The match stimulus for each trial was selected based on a staircase procedure from a set of many possible match stimuli that was generated and stored prior to the experiments. The match stimuli ranged across 149 logarithmically spaced regularity levels from very regular (0.097 jitter arcmin) to very irregular (154.24 jitter arcmin) plus a perfectly regular level (0 jitter arcmin).

### Design and Procedure

Two of the authors (Observers 1 & 2) and three naïve observers (Observers 3, 4 & 5) participated in the experiment, all having normal or adjusted-to-normal vision. All participants gave written informed consent before taking part in the experiment. The experimental protocols were approved by the Research Ethics Board (REB) of the Research Institute of McGill University Health Center (RI-MUHC) and conducted in accordance with their guideline. Each observer completed 125 trials (five independent staircases, each 25 trials) for each of the 18 conditions, in several sessions. In each session the observers completed several blocks, each containing three separate interleaved staircases (3 × 25 = 75 trials in each block) from three different conditions with the same center regularity to avoid potential adaptation from a different center regularity. The interleaved procedure helps prevent observers from ignoring the test before making a response and/or exerting any response bias to some conditions, because the test (both the surround and the appearance of the center) was changing trial by trial and the conditions on successive trials were unpredictable.

**Figure 3:**
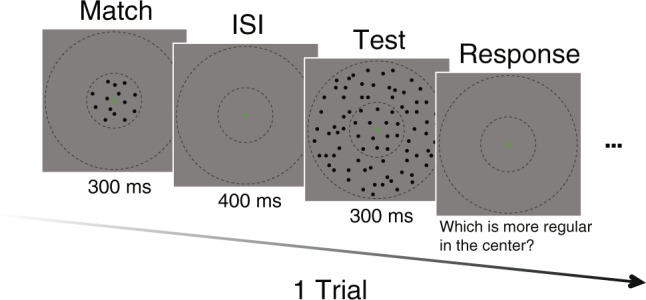
The stimulus presentation protocol for a trial. A match stimulus was presented first for 300 ms, followed by 400 ms ISI, and then a test stimulus was presented for 300 ms. Observers chose the more regular pattern between match and test stimuli in the center region using a numeric keypad. A green fixation mark was shown throughout the experiment. The dashed circles indicated the center and surround regions, which were not visible in the actual experiment. This schematic depiction is for demonstration purposes, and the actual size and scale of stimuli are as described in ‘Methods’.

In each trial a match stimulus and a test stimulus were presented sequentially (300 ms each, separated by a 400 ms interstimulus interval, see Fig. 3). We presented the match before the test to prevent potential adaptation from the test surround^31^. Observers pressed buttons on a numeric keypad to indicate which stimulus appeared to have a more regular pattern in the center region, with no time limit and no feedback. Each succeeding trial started 600 ms after the response to the preceding one. A green fixation cross (8.76 arcmin in height and width) was always presented in the middle of the center area, to insure alignment of the retinal images of the center areas between the test stimulus and the match. We also applied a small jitter (9.73 arcmin range, in horizontal and vertical directions, independently) on the fixation target for each stimulus to avoid potential afterimages, which might otherwise occur when the texture is highly regular. Observers were instructed to fixate throughout the experiment and respond only to the perceived regularity of the center areas regardless of the surround. The center-surround boundary was indicated by a red circle in practice trials, but it was removed in the formal experiment.

We used a 1-up-1-down staircase to estimate a point of subjective equality (PSE) in this appearance judgment^36^ for each condition, with each staircase conducted independently. The regularity level of the match in the first trial of each staircase was randomly chosen in a range bracketing 1–5 levels higher or lower than the physical regularity level of the test center. The jump size (regularity difference) of subsequent match trials in a staircase was gradually reduced from 18 to 3 levels in the first 16 trials, and remained constant in the later trials to get convergence.

### Data analysis

Data analysis was the same as in our previous studies^31,37^. Responses to the 125 trials in each condition were first summed across the five staircases. The responses of logarithmically spaced regularity levels were then log-transformed before fitting a logistic psychometric function (PF) according to a maximum likelihood criterion, using functions from the Palamedes Toolbox^38^. Points of subjective equality (PSEs) and slopes were estimated from the fits and then transformed back to linear-scale values for plotting in graphs. Standard errors (calculated as standard deviations) for PSE and slope of each condition were estimated by a bootstrap analysis^39^ in Palamedes, based on 400 sets of hypothetical data re-sampled from the collected data^36^. Group data were calculated by pooling the individual PSE and slope values across the five observers and calculating their geometric means and geometric standard errors for each condition.

## Results

**Figure 4:**
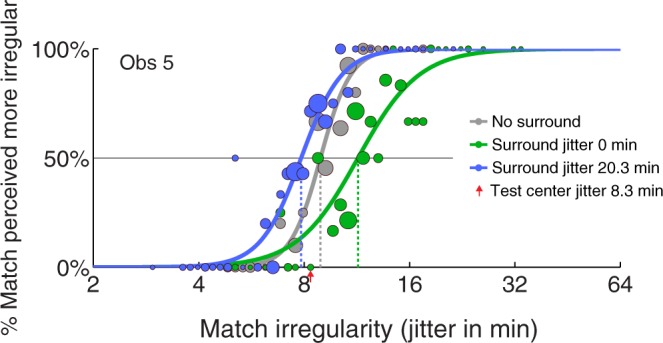
Psychometric functions (PFs) of three experimental conditions for a naïve observer. Each filled circle plots the proportion of times the match patch appears more irregular than the test patch in the center area under a match regularity level. The diameters of the circles correspond to the number of trials tested with that value of match density, as determined by the staircase procedure. Continuous lines (PFs) are best fitting logistic functions to the filled circles. Physical test center regularities were all 8.34 jitter arcmin (red arrow) while test surround regularities were 0 jitter arcmin (green line & circles) and 20.31 jitter arcmin (blue line & circles). No-surround baseline is shown as grey line & circles. The points of subjective equality (PSEs) are where the vertical dashed lines meet the abscissae and correspond to the 50% horizontal line.

PFs and PSEs for each condition were calculated from the data collected for each observer. Three example PFs for a naive observer (Observer 5) are shown in Fig. 4 - each one represents the proportion of times the match patch appears more irregular than the test patch under a match regularity level. Test center regularities were all 8.34 jitter arcmin (indicated by the red arrow), and test surround regularities were 0 jitter arcmin (green line & circles) and 20.31 jitter arcmin (blue line & circles). Responses from the no-surround baseline are shown by the grey line and circles. The PFs (continuous lines) are best-fitting logistic functions for the filled circles. The size of each circle corresponds to the number of trials presented at that match regularity level, which tend to be concentrated around the PSEs due to the nature of the staircase procedure. The PSEs are estimated from the PFs at the point where the vertical dashed lines meet the abscissae, corresponding to the 50% horizontal line. As shown in Fig. 4, the more regular surround PF (green) is shifted to the right of, and the less regular surround PF (blue) shifted to the left of, the baseline PF (grey), indicating the bidirectionality of SRC for this observer at the particular test center regularity. This result is consistent with the previous findings of bidirectional regularity/randomness aftereffect^7^.

**Figure 5:**
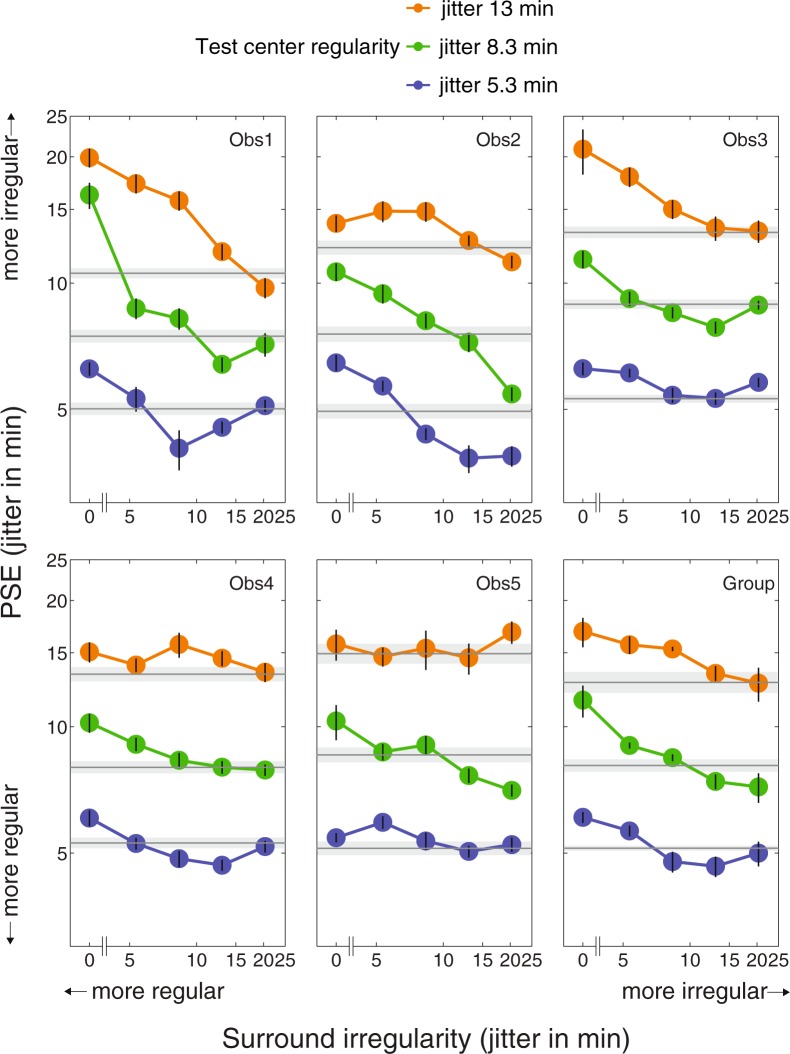
Individual and group PSE results. Graphs showing individual PSE data as a function of the absolute surround regularity levels for the five observers and their group average. Blue, green and orange lines represent the three test center regularities (5.34, 8.34 and 13.01 jitter arcmin) respectively. No-surround baselines of the three center regularities indicated by grey lines. Error bars (for no-surround conditions the light grey areas) of individual data are bootstrap-estimated standard errors. For the group data the geometric mean PSEs and geometric standard errors calculated across the five observers are shown in the last graph.

The PFs and PSEs of all the 18 conditions were calculated in the same way for all observers. The full set of PSE values are plotted as a function of the test surround regularity levels in Fig. 5, for test center regularities 5.34 jitter arcmin (blue), 8.34 jitter arcmin (green) and 13.01 jitter arcmin (orange), with no-surround baselines shown in grey. The group-average PSEs, across the five observers, are shown in the bottom right panel of Fig. 5. PSEs above the grey baselines imply an enhancement in perceived irregularity (i.e. reduction in perceived regularity), while below-baseline PSEs imply a decline in perceived irregularity (i.e. increase in perceived regularity). In general, the left part of the graphs (more regular surrounds) shows the former pattern and the right part (less regular surrounds) shows the latter. Such a pattern of results is indicative of a bidirectional SRC effect, such that more regular surrounds reduce perceived regularity of the center texture while more irregular surrounds enhance it. Interestingly, the reduction in perceived regularity can be observed in most conditions (e.g. shown in almost all of the three test center regularities) across observers, but the enhancement in perceived regularity is rather less robust (e.g. shown in only one of the three test center regularities, and only in some observers).

Note that in Fig. 5 the absolute regularity levels of the surround conditions are the same and are aligned across the three center regularities, however for each column of the PSE points, the center-surround *relative* regularities are different due to different center regularities. To better understand how the center-surround relative regularities affect SRC, we normalize the PSEs of each center regularity to their no-surround baseline PSE using the following formula:

$$SRC\,size=\frac{PSE\,of\,each\,condition-baseline\,PSE}{baseline\,PSE}\times 100 \% $$

**Figure 6:**
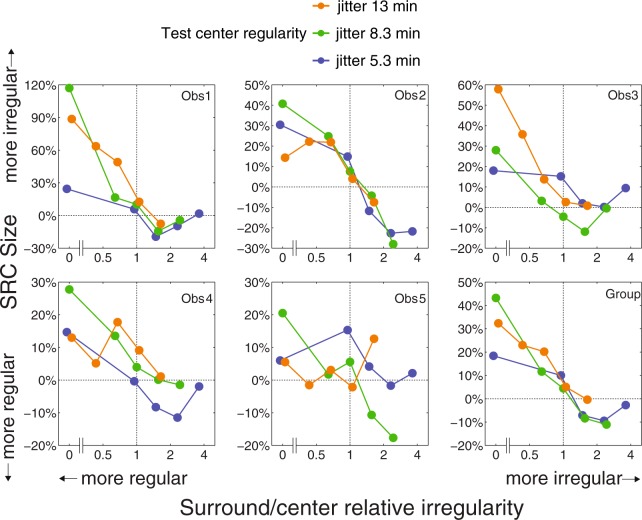
Normalized simultaneous regularity contrast (SRC) effect for each observer and their group average. All the PSEs in Fig. 5 were subtracted by the PSEs of their no-surround baselines then divided by the PSEs of the baselines. Therefore the y axis represents the percentage of perceived regularity change compared with the baselines. SRCs above the 0% horizontal dashed lines indicate increases in perceived irregularity, and SRCs below it indicate decreases (more regular). The x axis is plotted as a function of the relative regularity levels of the test surrounds and test centers (surround jitter min/center jitter min). Vertical dashed lines represent points where test center and surround regularities are physically equal, and left part represents more regular surrounds while right part represents more irregular surrounds. Blue, green and orange lines represent the three test center regularities (5.34, 8.34 and 13.01 jitter arcmin) respectively. The whole blue patterns are shifted to the left and the whole orange patterns are shifted to the right slightly to avoid overlapping. In general, more irregular surrounds enhance perceived regularity but more regular surrounds reduce it.

These normalized SRC values, representing the percentage change in perceived irregularity relative to the baseline, are plotted as a function of surround (jitter min)/center (jitter min) relative irregularity in Fig. 6 for each observer, and for their group average. Vertical dotted lines represent points where surround and test regularities are physically equal, with more regular surrounds to the left and less regular surrounds to the right. Positive SRCs (above the 0% horizontal dotted lines) indicate increases in perceived irregularity (less regular), and negative SRCs (below the 0% horizontal dotted lines) indicate decreases (more regular). Blue, green and orange lines represent the three test center regularities (5.34, 8.34 and 13.01 jitter arcmin), respectively. This figure shows that in general the more regular the surround compared to the center (left of the vertical dotted lines), the more irregular the center is perceived. On the other hand, a more irregular surround (right of the vertical dotted lines) usually makes the center appear more regular. Interestingly, this SRC effect seems asymmetric. That is, the enhancement of perceived irregularity is about 20–40%, but its decline (i.e. enhancement of perceived regularity) is only about 10% on average. Potential explanation for this asymmetry is further explored below.

## Discussion

We have demonstrated simultaneous regularity contrast, or SRC, consistent with previous studies of successive regularity contrast, i.e. of a regularity aftereffect^7,8^. The two aforementioned studies employed only one test regularity (positional jitter about half of the interelement distance) and in the case of the Yamada *et al*.^7^ study, only three adaptation levels (higher, lower or the same as the test regularity). Here we tested a wider range of test regularities and inducer (surround) regularities. The size of the SRC effect we observed is similar to that of the previously measured regularity aftereffect, i.e. around 18% on average^7^. However we also found individual differences in SRC directionality across the three test regularities, and asymmetric SRC directionality effects, as discussed as below.

### Directionality of SRC

In general our results indicate a bidirectional SRC effect, in that surrounds more regular than the center reduce perceived regularity while surrounds less regular than the center enhance it. This is consistent with the bidirectional regularity aftereffect in a previously reported study^7^, and confirmed in our own unpublished data.

In the present study there are however individual differences in directionality across the three test regularities. Some observers showed bidirectional SRC across all three test regularities (i.e. Observers 1& 2), while others showed bidirectionality only for mid or high test regularities (i.e. green and blue lines in Fig. 6 for Observers 3,4,5). The cause of these individual differences is unclear. For the lowest test regularity (orange lines in Fig. 6) the SRC bidirectionality is very weak, implying that the test center might be too irregular to be affected by a more irregular surround. This is the likely reason why Ouhnana *et al*.^8^ failed to find bidirectionality in their study of the regularity aftereffect: across a range of adaptor regularities they only employed a single, low test regularity, with a positional jitter level of 15 min.

### Asymmetry of SRC

As Fig. 6 shows, more-regular surrounds enhance perceived irregularity by 20% to 40%, while less-regular surrounds enhance perceived regularity by about 10%, an asymmetry seen across all observers. The regularity aftereffect observed in the previous study^7^, however was quite symmetric in that regular and irregular adaptors elicited similar sized but opposite shifts in perceived test regularity. That study used a stimulus with four times as many dots as our test stimulus and a larger interdot distance (46.8 min, which is double ours), allowing for a larger jitter range (41.4 min, again double ours) and hence more irregular adaptors. How the proportion between jitter range and interdot distance affects regularity perception is still unknown. On the other hand it is possible that an asymmetry in the aftereffect would have been observed if other adaptor/test regularity combinations had been tested. Another possible reason for the asymmetry, suggested by an anonymous reviewer, is that regular surrounds might attract more attention than irregular surrounds, leading to stronger SRC. Future studies are needed to test whether regular textures attract more attention than irregular textures. We will return to a brief discussion of the asymmetry found in the present study in the next section.

### Potential models for regularity encoding

While this study has been aimed at studying the nature of regularity encoding, one should consider the possibility that the psychophysical task might reflect something else entirely. In particular, note that in Fig. 2 the center with the more regular surround appears smaller and the center with the less regular surround appears larger - conceivably this perceived size effect might mediate our reported regularity effect. However in the formal experiment the stimuli were not presented side-by side as in Fig. 2. In the beginning of each experiment trial, a match stimulus was shown first, which should give a strong impression of the size of the center area for the following test stimulus. In addition, participants were instructed to always fixate at the center. Given such a rapid stimulus duration (300 ms), participants’ judgments about regularity should be largely based on the near-fixation area rather than the near-border area. Therefore the effect of area size should be very limited in this experiment. In addition, there is no direct or potential evidence showing that area size (not element size) can affect regularity perception. Consequently we believe this explanation unlikely, and instead interpret our results in terms of regularity coding.

Ouhnana *et al*.^8^ suggested that the regularity of a pattern might be encoded by the “peakedness” in the population response of channels selective for different ranges of spatial frequency (SF). This idea might seem counterintuitive, as it suggests that the information for encoding regularity lies in the Fourier (or wavelet) amplitude rather than phase spectrum, i.e. not primarily via a mechanism that explicitly encodes the local positional relationships in the stimulus. Ouhnana *et al*. based their reasoning on the observation that if one took a perfectly regular and a perfectly irregular pattern and swapped their Fourier amplitude and phase spectra, the resulting images revealed that regularity was preserved primarily in the amplitude spectrum. Subsequent studies of the regularity aftereffect^7^ and of regularity discrimination^18^ have also argued for SF-peakedness as the code for regularity. Moreover, different degrees of pattern regularity can be effortlessly segmented (see Fig. 2 here, also Fig. 1 of  ^8^), which places pattern regularity among the subset of texture dimensions that have traditionally been modelled using Fourier (or wavelet) energy-based operations, such as the conventional Filter-Rectify-Filter model^40,41^.

**Figure 7:**
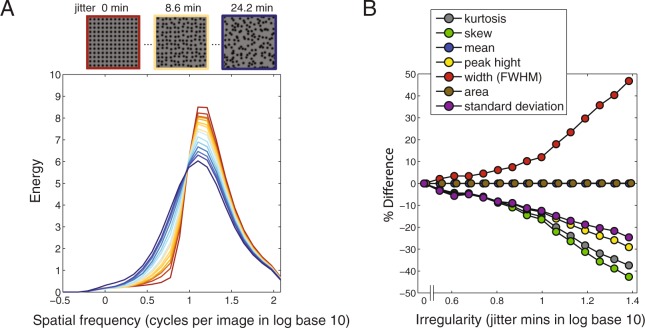
Gabor wavelet analysis results for stimulus images at fifteen regularity levels, as functions of spatial frequency. The stimulus images (256 × 256 pixels each, as shown in A, top) were filtered by odd-symmetric oriented Gabor filters with a wide range of spatial frequencies. Then the pixelwise root-mean-square responses (i.e. energy) of the filtered images were calculated and normalized by the square of the filter sizes, as plotted in (**A**), from high regularities (dark orange lines, which are sharper) to low regularities (dark blue lines, which are flatter). Each line represents averaged spatial frequency distribution across four orientations of Gabor filters (vertical, horizontal and ± 45 degree oblique) and nine sample images. For each distribution in (**A**), we calculated its kurtosis, skew, mean, peak height (maximum), width (full width at half maximum, FWHM), area under the curve, and standard deviation. The value of each statistic is calculated across the fifteen regularity levels and normalized to the value at the most regular condition (jitter 0 min). The plot in (**B**) shows the percentage difference compared with the jitter 0 min condition for each type of statistic. The width of spatial frequency distribution increases with irregularity, while kurtosis, skew, peak height and standard deviation decrease with it.

Here we re-examine the idea of SF-peakedness, as well as consider other possibilities. To illustrate how regularity might in principle be computed from SF-peakedness, we performed a similar SF analysis as Ouhnana, *et al*.^8^, for fifteen regularity levels of our stimuli (cut to 256 × 256 pixels each, as shown in Fig. 7A, top). Each image was filtered by a set of odd-symmetric Gabor filters ranging from low to high SFs (27 log-spaced SFs from 0.18 to 120.65 cycles per image, each 1.5 bandwidths in octaves), each at four orientations (0, 90 and ± 45 degree). The pixelwise root-mean-square (RMS) responses (i.e. energy) of each filtered image was then calculated and normalized by the square of the filter size. The normalized RMS values of each regularity level were then averaged across the four orientations and across nine sample images, normalized to equate total response across regularity levels (i.e. the area normalization). The results, plotted in Fig. 7A, show that as one proceeds from high (dark orange lines) to low regularity (dark blue lines) the SF distribution becomes less peaked, i.e. more spread out. Note that with our stimuli none of the SF distributions show two peaks, which had been found in previous studies^8,18^. This is because in our stimuli the peak associated with the element size and the peak associated with the element spacing overlap to give a composite peak at about 12 cycles/image (1.08 log cycles/image). The SF distributions in Fig. 7A were averaged across four orientations since the pattern is generally the same, i.e. that it becomes less peaked with irregularity whether the filter orientation aligns with the grid orientation (0 and 90 degree) or not (±45 degree).

SF-peakedness is a rather loosely-defined term, so the question arises as to what might be an appropriate metric to embody it. There are a number of possibilities, for example kurtosis, peak height (maximum), width at half maximum (FWHM), and standard deviation. To compare these statistics, along with other non-peakedness statistics such as skew, area and mean, we normalized each statistic to that of the most regular pattern (jitter 0 min) and then plotted the percentage difference in the statistic as a function of irregularity. As shown in Fig. 7B, the width (FWHM) of the SF distribution increases with irregularity, while kurtosis, skew, peak height and standard deviation all decrease with it. The area and mean values remain constant across irregularity due to the area normalization. All of the statistics that covary with regularity could be potential candidates for coding regularity. For example, peak height, the precise statistic proposed in the aforementioned studies on regularity^7,8,18^ is a good predictor of regularity. However other statistics such as width, kurtosis, skew and standard deviation might also be important - the first of the three appear to be even more sensitive to the degree of irregularity than peak height (Fig. 7B). However skew might not be a reliable source of regularity estimation since it is largely determined by the relationship between element size and element spacing. For example, the main peak of a SF distribution associates with the element size and the second peak associates with the element spacing. In the previous studies element spacing is larger than element size, therefore the second peak is at the left of the main peak^8,18^. In this case skew decreases with regularity. However if element spacing is smaller than element size, the second peak will move to the right of the main peak. In this case skew increases with regularity. Therefore skew might not be a good predictor of regularity, and future studies are needed to test for this.

**Figure 8:**
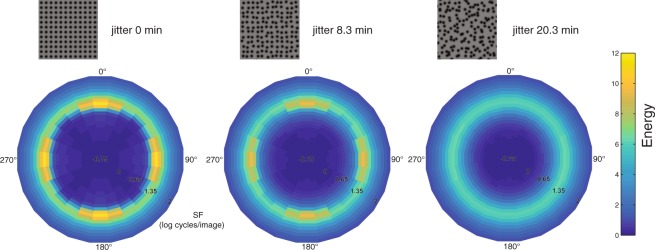
Gabor wavelet analysis results for stimulus images of three regularity levels (0, 8.3 and 20.3 jitter min) shown as polar image plots of average energy (calculated as for Fig. 7A) as a joint function of spatial frequency (distance from center) and orientation (polar angle). The polar images are generated using the Matlab function ‘polarPcolor’ created by Etienne Cheynet (www.mathworks.com/matlabcentral/fileexchange/49040-pcolor-in-polar-coordinates). Average energy responses were obtained across a broad range of spatial frequencies, and at each of 12 orientations of Gabor filters (0–165 degrees, with a 15-degree spacing, and 180 deg-opposite values re-plotted symmetrically). Higher energy responses are concentrated around the grid orientations (vertical and horizontal) and peak SF (1.08 log cycles/image) for regular texture (left), with the energy gradually spreading out across orientations with increasing irregularity (middle to right).

Mark Georgeson (personal communication) has suggested that regularity might be coded not by the pattern of responses across SF but instead across orientation. Indeed, Yamada, *et al*.^7^ showed that the regularity aftereffect was sensitive to the orientation relationship between the adaptor and test dot grids, consistent with orientation being important for coding regularity. To examine this, we performed the same SF analysis as before for three selected regularities (0, 8.3 and 20.3 jitter min) but plotted the data separately for a wide range of Gabor filter orientations (0–165 degree, with a 15-degree interval). As shown in Fig. 8, the high energy responses are concentrated around the grid orientations (vertical and horizontal) for regular textures (Fig. 8 left), and the energy gradually spreads out across orientation with increasing irregularity (Fig. 8 right). Thus as Georgeson suggests, the variance of the distribution of energy across orientation (and other statistics such as width, kurtosis and skew) could also code regularity.

Notwithstanding the precise statistic that the visual system uses to code for regularity, how might we explain SRC? We envision two possible kinds of approaches. One is that the surround-test relationship acts directly on the regularity statistic. If so, the bidirectionality in SRC would imply that the code for regularity is represented in an opponent manner, with one pole for ‘regular’ the other ‘irregular’, analogous to the ‘red-green’ and ‘blue-yellow’ opponency in color appearance^42^. Suppression of the regular pole in the center by a regular surround would act to shift the position of the center’s regularity towards the irregular pole, whilst suppression of the irregular pole by an irregular surround would do the opposite, just as a red surround causes a center grey to appear greenish and a green surround influences a center grey to appear reddish. So for example if kurtosis (*k*) of the SF distribution was the code for regularity, the following formulation might apply:

$${\rm{perceived}}\,{\rm{center}}\,k={\rm{physical}}\,{\rm{center}}\,k\times (\frac{{\rm{physical}}\,{\rm{center}}\,k}{{\rm{physical}}\,{\rm{surround}}\,k}\times 100 \% )$$

**Figure 9:**
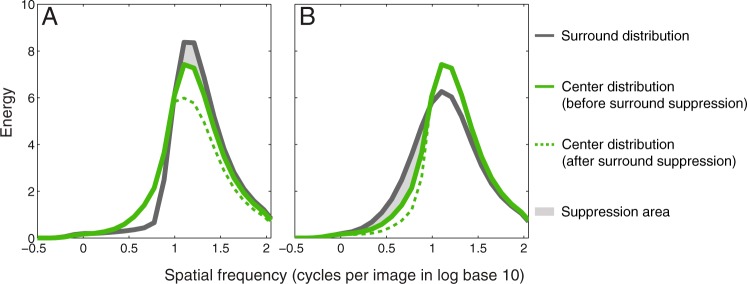
Examples of channel-selective simultaneous contrast-contrast approach to explain SRC. Solid green lines in both graphs show the SF distribution with center regularity 8.34 jitter arcmin without surround suppression, using the same analysis as Fig. 7A. The grey lines represent the SF distribution of a more regular surround (**A**, 0 jitter arcmin) and a less regular surround (**B**, 20.31 jitter arcmin). Dotted green lines represent the center distribution after surround suppression though simultaneous contrast-contrast. The regular surround (**A**) and irregular surround (**B**) suppress center responses in different SF bands (grey areas). As a result, the center distribution becomes flatter, and therefore is perceived as more irregular in the presence of a more regular surround (**A**). However in (**B**) the center distribution becomes sharper and therefore is perceived as more regular in the presence of a more irregular surround.

A second approach towards understanding SRC might be that the individual SF (or orientation) channels in the surround inhibit those in the centre via channel-selective simultaneous “contrast-contrast”^43^. The idea is illustrated in Fig. 9 for the SF channel approach. An intriguing aspect of this idea is that even though simultaneous contrast-contrast might be acting unidirectionally, i.e. the surround works only to reduce the center channel responses, the consequence is a bidirectional SRC. The reason for this is that regular and irregular surrounds suppress center responses of (somewhat) different SF bands. A regular surround suppresses the higher SF bands of the center distribution due to the higher contrast energy of the surround compared to the center, in the higher SF bands (Fig. 9A). As a result, the center distribution becomes flatter and therefore it is perceived as more irregular in the presence of a regular surround. On the other hand, an irregular surround suppresses the lower SF bands of the center distribution (Fig. 9B), so it becomes sharper and therefore is perceived as more regular.

Our informal simulation modeling based on the opponency and SF-selective contrast-contrast approaches described above shows that both models can qualitatively predict the pattern of SRC we have observed, including the asymmetry described earlier. However, given the possible number of regularity-code statistics, and the possible model approaches to the SRC outlined above, it seems premature to commit ourselves to a specific model of the SRC at this stage. An important future direction will be to undertake new experiments aimed at explicitly testing the various model approaches described here.

The two above approaches to explaining SRC are based on separate SF analyses for the center and surround regularities, not on whole-stimulus SF analyses, since the aim was to capture the lateral inhibitory interactions presumed to underpin SRC. Also note that the models we have proposed are preliminary in the sense that they have not considered how regularity is encoded in three dimensions (3D). It is for example possible that regularity information is encoded independently of 3D viewing angle^44^ - in other words, the image statistics relevant to regularity (e.g. kurtosis of SF distribution) might be adjusted or normalized for viewing angle/slant. Future studies are needed to test this idea. Moreover, the regularity models discussed here are focused on 2D-grid patterns and may not be applicable to classic symmetries and other types of stimuli, for example very dense textures consisting of multiple spatial scales of luminance variation. Further studies are therefore needed to determine the image statistics important to other types of stimuli exhibiting regularity.

## Conclusion

In this study we have demonstrated simultaneous regularity contrast (SRC) and shown that it is bidirectional and asymmetric. Regular surrounds have a stronger influence on the perception of center regularity than irregular surrounds. Analysis of the spatial frequency distributions of pattern regularity using multiple narrowband, oriented Gabor filters reveals that increasing irregularity is accompanied by a flattening of the response distribution. We suggest that in addition to “peakedness” of spatial frequency distributions, other statistics such as kurtosis and width that can depict this shape change could also serve as candidate codes for regularity in the visual system. Finally we suggest that the SRC might be mediated either by a regular-irregular spatial opponency mechanism, or by a contrast-contrast mechanism operating separately on spatial-frequency and/or orientation channels.

## References

[1] Gibson JJ (1950). The Perception of Visual Surfaces. The American Journal of Psychology.

[2] Lin, W.-C., Hays, J. H., Wu, C., Liu, Y. & Kwatra, V. In *2006 IEEE Computer Society Conference on Computer Vision and Pattern Recognition (CVPR'06)* Vol. 1, 427–434 (2006).

[3] Knill DC (1998). Surface orientation from texture: ideal observers, generic observers and the information content of texture cues. Vision Research.

[4] Protonotarios, E. D., Baum, B., Johnston, A., Hunter, G. L. & Griffin, L. D. An absolute interval scale of order for point patterns. *Journal of The Royal Society Interface* **11** (2014). 10.1098/rsif.2014.0342 PMC4233723 25079866

[5] Gibson, J. J. *The perception of the visual world*. (Riverside Press, 1950).

[6] Protonotarios ED, Johnston A, Griffin LD (2016). Difference magnitude is not measured by discrimination steps for order of point patterns. Journal of Vision.

[7] Yamada Y, Kawabe T, Miyazaki M (2013). Pattern randomness aftereffect. Scientific Reports.

[8] Ouhnana M, Bell J, Solomon JA, Kingdom FAA (2013). Aftereffect of perceived regularity. Journal of Vision.

[9] Kohler PJ, Cottereau BR, Norcia AM (2018). Dynamics of perceptual decisions about symmetry in visual cortex. NeuroImage.

[10] Kohler PJ, Clarke A, Yakovleva A, Liu Y, Norcia AM (2016). Representation of Maximally Regular Textures in Human Visual Cortex. The Journal of Neuroscience.

[11] van der Helm PA (2010). Weber-Fechner behavior in symmetry perception?. Attention, Perception, & Psychophysics.

[12] Csathó A, van der Vloed G, van der Helm PA (2004). The force of symmetry revisited: symmetry-to-noise ratios regulate (a)symmetry effects. Acta psychologica.

[13] Makin ADJ (2016). An Electrophysiological Index of Perceptual Goodness. Cerebral Cortex.

[14] Jennings B, Kingdom F (2018). Different symmetries, different mechanisms. Journal of Vision.

[15] Emrith K, Chantler MJ, Green PR, Maloney LT, Clarke ADF (2010). Measuring perceived differences in surface texture due to changes in higher order statistics. J. Opt. Soc. Am. A.

[16] Morgan MJ, Mareschal I, Chubb C, Solomon JA (2012). Perceived pattern regularity computed as a summary statistic: implications for camouflage. Proceedings of the Royal Society B: Biological Sciences.

[17] Hess RF, Barnes G, Dumoulin SO, Dakin SC (2003). How many positions can we perceptually encode, one or many?. Vision Research.

[18] Protonotarios, E. D., Michael L, Alan, J. & Griffin, L. D. In *40th European Conference on Visual Perception (ECVP)* (Berlin, Germany, 2017).

[19] Ginsburg N (1976). Effect of Item Arrangement on Perceived Numerosity: Randomness vs Regularity. Perceptual and Motor Skills.

[20] Dakin SC, Tibber M, Greenwood JA, Kingdom FAA, Morgan MJ (2011). A common perceptual metric for human discrimination of number and density. Proceedings of the National Academy of Sciences.

[21] Vancleef K (2013). Spatial arrangement in texture discrimination and texture segregation. i-Perception.

[22] Machilsen B, Wagemans J, Demeyer M (2016). Quantifying density cues in grouping displays. Vision Research.

[23] Klein S, Stromeyer CF, Ganz L (1974). The simultaneous spatial frequency shift: A dissociation between the detection and perception of gratings. Vision Research.

[24] Chubb C, Sperling G, Solomon JA (1989). Texture interactions determine perceived contrast. Proceedings of the National Academy of Sciences of the United States of America.

[25] Georgeson MA (1985). The effect of spatial adaptation on perceived contrast. Spatial Vision.

[26] Heinemann EG (1955). Simultaneous brightness induction as a function of inducing-and test-field luminances. Journal of experimental psychology.

[27] Blakemore C, Carpenter RHS, Georgeson MA (1970). Lateral inhibition between orientation detectors in the human visual system. Nature.

[28] Clifford CWG (2014). The tilt illusion: Phenomenology and functional implications. Vision Research.

[29] Roberts B, Harris MG, Yates TA (2005). The Roles of Inducer Size and Distance in the Ebbinghaus Illusion (Titchener Circles). Perception.

[30] Mackay DM (1973). Lateral interaction between neural channels sensitive to texture density?. Nature.

[31] Sun H-C, Baker JCL, Kingdom FAA (2016). Simultaneous density contrast is bidirectional. Journal of Vision.

[32] Brainard DH (1997). The Psychophysics Toolbox. Spatial Vision.

[33] Pelli DG (1997). The VideoToolbox software for visual psychophysics: transforming numbers into movies. Spatial Vision.

[34] Kleiner M, Brainard D, Pelli D (2007). What’s new in Psychtoolbox-3?. Perception.

[35] Ban H, Yamamoto H (2013). A non–device-specific approach to display characterization based on linear, nonlinear, and hybrid search algorithms. Journal of Vision.

[36] Kingdom, F. A. A. & Prins, N. *Psychophysics: A Practical Introduction*, *Second Edition* (Academic Press, 2016).

[37] Sun H-C, Kingdom FAA, Baker JCL (2017). Texture density adaptation can be bidirectional. Journal of Vision.

[38] Prins, N. & Kingdom, F. A. A. Palamedes: Matlab routines for analyzing psychophysical data. http://www.palamedestoolbox.org/ (2009).

[39] Efron, B. & Tibshirani, R. J. *An introduction to the bootstrap*. (CRC press, 1994).

[40] Graham NV (2011). Beyond multiple pattern analyzers modeled as linear filters (as classical V1 simple cells): Useful additions of the last 25 years. Vision Research.

[41] Landy, M. S. In *The New* Visu*al* Neur*osciences* (eds Werner, J. S. & Chalupa, L. M.) Ch. 45, 639–652 (MIT Press, 2013).

[42] Hering, E. *Outlines of a theory of the light sense*. (Harvard University Press, 1964).

[43] Cannon MW, Fullenkamp SC (1993). Spatial interactions in apparent contrast: Individual differences in enhancement and suppression effects. Vision Research.

[44] Makin ADJ, Rampone G, Bertamini M (2015). Conditions for view invariance in the neural response to visual symmetry. Psychophysiology.

